# The complete chloroplast genome of *Antiaris toxicaria*, a medicinal and extremely toxic species

**DOI:** 10.1080/23802359.2018.1516121

**Published:** 2018-09-10

**Authors:** Lushui Zhang, Xiaoyue Yang, Xingxing Mao, Zefu Wang

**Affiliations:** a Key Laboratory of Bio-Resource and Eco-Environment of Ministry of Education College of Life Sciences, Sichuan University, Chengdu, China;; b Ecological Security and Protection Key Laboratory of Sichuan Province, Mianyang Normal University, Mianyang, China

**Keywords:** Complete chloroplast genome, *Antiaris toxicaria*, phylogenetic analysis

## Abstract

*Antiaris toxicaria* (*Antiaris*, Moraceae) is a medicinal and extremely toxic species. To facilitate species identification and provide genetic information, we determined the complete chloroplast genome of *A. toxicaria* using the Illumina platform. The genome was 161,412 bp in length, comprising a large single-copy region (LSC) of 89,883 bp, a small single-copy region (SSC) of 20,375 bp, and a pair of inverted repeats (IRs) of 25,577 bp each. The genome contained 130 encoded genes in total, including 85 protein-coding genes, 8 ribosomal RNA genes, and 37 transfer RNA genes. The overall GC content of the *A. toxicaria* chloroplast genome is 35.87%. The phylogenetic analysis revealed *A. toxicaria* was closely related to the genus *Ficus* within the family Moraceae.

*Antiaris toxicaria* (*Antiaris*, Moraceae) is a medicinal and extremely toxic species. It is said that the utilization of *A. toxicaria* began in the 19th century. However, there is still rare genetic information of *A. toxicaria*. Herein, we reported the chloroplast genome of *A. toxicaria*. The annotated chloroplast genome has been submitted to GenBank under the accession number of MH606237.

We collected the fresh leaves of an *A. toxicaria* individual from Jinghong in Yunnan Province, China (100 °54′E, 21 °48′N). Voucher specimen of the species was stored in the Key Laboratory of Bio-resource and Eco-environment of Ministry of Education (Sichuan, China). The total DNA was extracted with the DNAsecure Plant Kit (TIANGEN). The whole-genome sequencing was carried out with the Hiseq4000 Platform (Illumina, USA). Finally, we obtained ∼10G high-quality base pairs of raw data. The software Bwa (Li 2013) and Samtools (Li et al. 2009) were used to map the Illumina reads to the reference *Morus indica* (DQ226511.1, Ravi et al. 2006) chloroplast genome. The mapped reads were extracted and then used to assemble the genome with the software NOVOPlasty v2.5.9 (Dierckxsens et al. 2017). We aligned the resulting contigs to the reference genome with Bwa and Samtools again and generated a complete genome by Genious v 11.1.14 (Kearse et al. 2012). Finally, we used GapCloser (Luo et al. 2012) to fill the gaps and obtain a chloroplast genome of *A. toxicaria* with a length of 161,412 bp. The annotation was performed with the software Plann (Huang and Cronk 2015) and Sequin (NCBI website).

The complete chloroplast genome of *A. toxicaria* contains two inverted repeat (IRA and IRB) regions with the length of 20,577 bp each, separated by a large single-copy (LSC) region of 89,883 bp and a small single-copy (SSC) region of 20,375 bp, respectively. The genome contains 130 genes, including 85 protein-coding genes (PCGs), 37 transfer RNA (tRNA) genes and 8 ribosomal RNA (rRNA) genes. Most of these genes are single copy genes, while 17 genes (6 PCGs, 7 tRNA genes, and 4 rRNA genes) were duplicated in the IR regions. The overall GC content of *A. toxicaria* chloroplast genome is 35.87%, and the LSC, SSC, and IR regions occupy 33.46%, 28.97% and 42.86%, respectively.

To infer the phylogenetic position of *A. toxicaria* and estimate the phylogenetic relationships within the family Moraceae, we reconstructed a phylogenetic tree based on the complete chloroplast genome sequences of 13 species including *A. toxicaria*. The sequences were aligned with the software MAFFT (Katoh and Standley 2013). And the phylogenetic tree was constructed using the software RaxML (Stamatakis 2014) with 100 bootstrap replicates based on the maximum likelihood (ML) method. The ML tree revealed *A. toxicaria* was closely related to the genus *Ficus* (Moraceae) with strongly support (Figure 1). Above all, we provide a valuable genomic information of *A. toxicaria*, which could help us identify and utilize this medicinal and extremely toxic.

**Figure 1.**
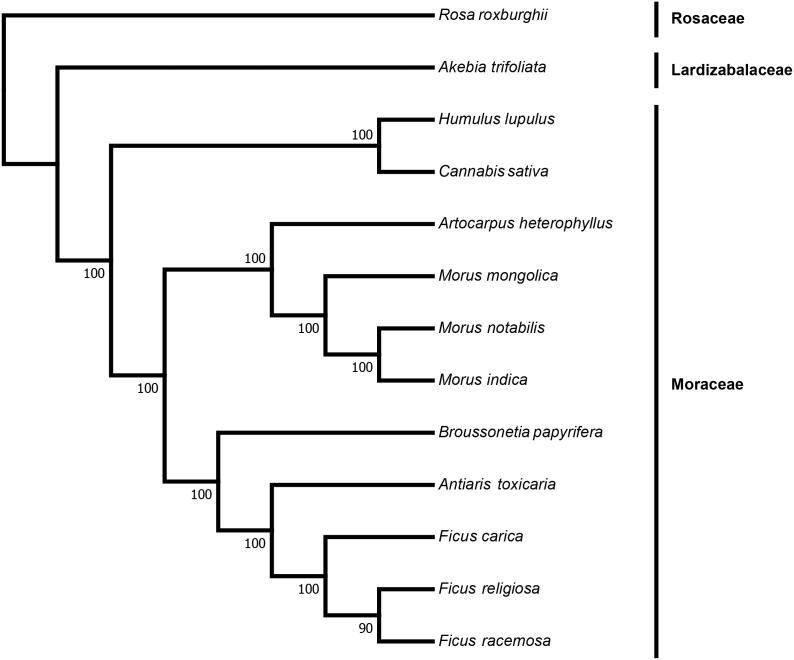
Maximum likelihood (ML) phylogenetic tree based on the complete chloroplast genome sequences of *Antiaris toxicaria* and other 12 species. Numbers in the nodes are the bootstrap values from 100 replicates. Their accession numbers are as follows: *Rosa roxburghii*: NC_032038.1, *Akebia trifoliata*: NC_029427.1, *Humulus lupulus*: NC_028032.1, *Cannabis sativa*: NC_026562.1, *Artocarpus heterophyllus*: MG434693.1, *Morus mongolica*: NC_025772.2, *Morus notabilis*: NC_027110.1, *Morus indica*: DQ226511.1, *Broussonetia papyrifera*: NC_035569.1, *Ficus carica*: NC_035237.1, *Ficus religiosa*: NC_033979.1, *Ficus racemosa*: NC_028185.1.

## References

[CIT0001] Dierckxsens N, Mardulyn P, Smits G 2017 Novoplasty: de novo assembly of organelle genomes from whole genome data. Nucleic Acid Res. 4(4):e18. 10.1093/nar/gkw955 PMC5389512 28204566

[CIT0002] Huang DI, Cronk QCB 2015 Plann: a command-line application for annotating plastome sequences. Appl Plant Sci. 3:1500026. 10.3732/apps.1500026 PMC4542940 26312193

[CIT0003] Katoh K, Standley DM 2013 MAFFT multiple sequence alignment software version 7: improvements in performance and usability. Mol Biol Evol. 30:772–780. 23329690 10.1093/molbev/mst010 PMC3603318

[CIT0004] Kearse M, Moir R, Wilson A, Stones-Havas S, Cheung M, Sturrock S, Buxton S, Cooper A, Markowitz S, Duran C, et al. 2012 Geneious basic: an integrated and extendable desktop software platform for the organization and analysis of sequence data. Bioinformatics. 28:1647–1649. 22543367 10.1093/bioinformatics/bts199 PMC3371832

[CIT0005] Li H 2013 Aligning sequence reads. Clone Sequences and Assembly Contigs with Bwa-Mem. 1303. arXiv preprint arXiv:1303.3997.

[CIT0006] Li H, Handsaker B, Wysoker A, Fennell T, Ruan J, Homer N, Marth G, Abecasis G, Durbin R 2009 The sequence alignment/map format and SAMtools. Bioinformatics. 25:2078–2079. 19505943 10.1093/bioinformatics/btp352 PMC2723002

[CIT0007] Luo R, Liu B, Xie Y, Li Z, Huang W, Yuan J, et al. 2012 Soapdenovo2: an empirically improved memory-efficient short-read de novo, assembler. Giga Sci. 1:1–6. 10.1186/2047-217X-1-18 PMC3626529 23587118

[CIT0008] Ravi V, Khurana JP, Tyagi AK, Khurana P 2006 The chloroplast genome of mulberry: complete nucleotide sequence, gene organization and comparative analysis. Tree Genet Genome. 3:49–59.

[CIT0009] Stamatakis A 2014 RAxML version 8: a tool for phylogenetic analysis and post-analysis of large phylogenies. Bioinformatics. 30:1312–1313. 24451623 10.1093/bioinformatics/btu033 PMC3998144

